# Post-COVID-19 Steroid-Induced Avascular Necrosis of the Hip: Does Severity of Infection Influence Functional Outcome?

**DOI:** 10.7759/cureus.88428

**Published:** 2025-07-21

**Authors:** Mobinul Hoque, Mohammad Lockman, Mohammad Kamruzzaman, Mohammad Mamun-or-Rashid, SM Moshee-ur-Rahman, Rabin C Halder, Rahman M Kazi, Akter Afrin, Fabliha Fyrose Ahmed, Sonia Akber

**Affiliations:** 1 Orthopaedic Surgery, National Institute of Traumatology and Orthopaedic Rehabilitation (NITOR), Dhaka, BGD; 2 Paediatric Orthopaedic Surgery, National Institute of Traumatology and Orthopaedic Rehabilitation (NITOR), Dhaka, BGD; 3 Nephrology, Shaheed Suhrawardy Medical College and Hospital, Dhaka, BGD; 4 Epidemiology and Biostatistics, Centre for Medical Research and Development (CMRD), Dhaka, BGD

**Keywords:** avascular necrosis, corticosteroid, covid-19, functional outcome, modified harris hip score, total hip replacement

## Abstract

Background: Corticosteroids are widely used in the treatment of moderate and severe coronavirus disease 2019 (COVID-19), but their association with avascular necrosis (AVN) of the femoral head has raised concern. This study aimed to evaluate the association between the severity of COVID-19 and functional outcome in patients undergoing total hip replacement (THR) for post-COVID-19 steroid-induced AVN.

Methods: This prospective observational study was conducted on 26 patients diagnosed with steroid-induced AVN of the femoral head following moderate or severe COVID-19 infection. All patients underwent cementless THR. The severity of COVID-19, steroid exposure, time to symptom onset, and preoperative and postoperative hip function were recorded. Functional outcome was assessed using the modified Harris hip score (MHHS) at 12 months.

Results: Patients with severe COVID-19 had a significantly higher total dose and longer steroid exposure (mean 2759.1 mg, 25.76 days) and earlier onset of hip pain (mean 54.1 days) than those with moderate infection (mean 1248.8 mg; 16.8 days, and 81.2 days, respectively). Both groups showed significant improvement in postoperative MHHS, with the moderate group achieving slightly higher scores (mean 89.2 vs. 85.8). The severity of AVN, total steroid dose, and duration had no significant association with outcome.

Conclusion: While THR provided satisfactory outcomes in all cases, patients with moderate COVID-19 exhibited marginally better functional recovery, suggesting that infection severity and steroid burden may influence postoperative prognosis.

## Introduction

The coronavirus disease 2019 (COVID-19), caused by the novel SARS-CoV-2, was first identified in Wuhan, China, in December 2019 [1]. Following its rapid global spread, the WHO declared it a pandemic on March 11, 2020 [2]. As of December 2024, there are 2,051,565 confirmed COVID-19 cases in Bangladesh, which is the second most affected country in South Asia after India [3]. COVID-19 has since posed unprecedented challenges to healthcare systems worldwide, prompting the adoption of various therapeutic strategies to mitigate its severe respiratory and systemic complications [2]. Among these, systemic corticosteroids, particularly dexamethasone and methylprednisolone, emerged as cornerstone treatments for moderate to severe COVID-19, primarily for their ability to suppress the hyperinflammatory 'cytokine storm' and reduce mortality in hospitalized patients [4,5]. Oral (60 to 80 mg) and injectable (250 mg) methylprednisolone were recommended in the national guideline for moderate and severe COVID cases, respectively [6].

About 8% to 10% of the steroid users later developed steroid-induced AVN, the mechanism of which remains uncertain [7]. Some studies stated that AVN develops in patients receiving long courses of steroids, while other literature suggests that the magnitude rather than frequency is more important [8-11]. Agarwala et al. even described AVN as a part of “long COVID-19” [12].

In severe AVN, total hip replacement (THR) is indicated to relieve pain and improve functional capabilities for the patient to lead a normal life [13]. However, the severity of COVID-19's impact on postoperative functional outcome in patients with post-COVID-19 steroid-induced AVN of the hip is still unexplored. Only Li et al. documented that 32% of the patients developed AVN after being treated for severe COVID-19 infection [14]. National scientific evidence is far scarcer. So, the present study aimed to assess if COVID-19 severity had any influence over post-surgical functional outcome in patients with post-COVID-19 steroid-induced AVN of the hip and gauge the percentage of patients achieving satisfactory recovery after surgery despite having steroid-induced AVN.

## Materials and methods

Study setting, design, and study participants

This prospective observational study was conducted at the National Institute of Traumatology and Orthopaedic Rehabilitation (NITOR), Dhaka, Bangladesh, from January 2023 to December 2024. The NITOR is the premier national-level tertiary care institute dedicated to the management of orthopaedic, trauma, and rehabilitation services. The center specializes in orthopaedic surgery, trauma management, and rehabilitation. It also functions as a leading teaching and research institute affiliated with the University of Dhaka. Ethical clearance was obtained from the Institutional Review Board of NITOR (approval no. 4237). All participants provided written informed consent, and patient confidentiality was maintained per the ethical standards outlined in the Declaration of Helsinki.

A total of 26 patients diagnosed with steroid-induced AVN of the femoral head were enrolled using a convenient sampling technique. Steroid-induced AVN was defined as “ischemic bone necrosis resulting from compromised blood supply to the femoral head, primarily due to corticosteroid therapy, leading to the death of bone tissue.” This condition is often associated with high-dose or prolonged corticosteroid use, which may alter lipid metabolism, induce fat embolism, and damage vascular endothelial cells, thereby impeding blood flow to the bone.

Inclusion criteria included patients of ≥18 years and of either sex, unilateral femoral head involvement, Ficat and Arlet class III and onwards, a documented history of moderate or severe COVID-19 infection, and corticosteroid therapy for a minimum of two weeks administered during COVID-19 illness. Exclusion criteria were AVN cases associated with corticosteroid use for non-COVID-19-related conditions; AVN due to non-steroidal or traumatic causes and bilateral hip involvement; patients with a BMI more than the normal range; diabetic, hypertensive, and hyperlipidemia patients with chronic kidney disease (CKD) and chronic obstructive pulmonary disease (COPD); and smokers and alcoholics. All cases meeting the above criteria and available during the study period at the study site were included. As the condition is relatively rare, no prior sample size calculation based on previous studies was performed.

Data collection 

The diagnosis of AVN was made clinically and radiologically, based on patient history, physical examination, and imaging (X-ray and MRI, where applicable). The AVN severity was assessed by the Ficat and Arlet classification from imaging. The severity of prior COVID-19 infection was classified according to the national clinical management guidelines. Moderate COVID-19 involved the presence of pneumonia on imaging with oxygen saturation (SpO₂) between 90% and 94% on room air. Severe COVID-19 meant SpO₂ < 90% on room air, respiratory rate > 30/min, or signs of severe respiratory distress, often requiring hospitalization and oxygen supplementation.

For each patient, the duration and dose of corticosteroid therapy administered during COVID-19 management were carefully documented. Only those who had received corticosteroids for at least 14 consecutive days were included in the study. In terms of steroid use, all moderate cases were subjected to oral steroids (72 to 80 mg for 14 to 21 days), and severe cases were administered oral (64 to 72 mg/day for 14 to 28 days) and injectable steroids (250 mg/day for five to seven days). The interval between cessation of steroid therapy and the onset of hip pain was recorded to assess the temporal relationship.

Surgical procedure

All patients underwent cementless THR based on clinical indication, using the direct lateral (Hardinge) approach under spinal anesthesia. Patients were positioned in the lateral decubitus position, and strict aseptic preparation was ensured. A lazy-J skin incision was made over the greater trochanter, the tensor fascia lata was retracted anteriorly and the gluteus maximus posteriorly, followed by splitting the gluteus medius, elevation of the vastus lateralis, and abduction of the thigh to expose the hip joint. The hip capsule was incised, and the femoral head was dislocated, and the femur retracted anteriorly. The neck was then cut at a 45° angle in the correct version. Acetabular preparation involved excision of the overlying capsule, labrum, reaming in concentric increments, and placement of a press-fit cementless cup at 45° inclination and 15° to 20° anteversion. A polyethylene liner was inserted after screw fixation. For the femoral component, reaming and broaching were performed with careful control of anteversion and axial stability. A hydroxyapatite-coated stem was then impacted, and a trial reduction was done to assess limb length and joint stability. After satisfactory alignment, the final femoral head was implanted, and soft tissues were repaired using non-absorbable sutures. The surgical implants used were a porous-coated acetabular cup, a polyethylene liner, a hydroxyapatite-coated femoral stem, and a ceramic head. The same combination of implants was used from the same manufacturer (DePuy Synthes, Raynham, MA, USA).

Follow-up

Postoperative care included early mobilization with walker support from the first postoperative day and full weight-bearing after six weeks. The primary outcome was assessment of hip function by a validated clinical assessment tool, the modified Harris hip score (MHHS), at 12 months following surgery. Score interpretation was categorized into four groups, namely excellent (90-100), good (80-89), fair (70-79), and poor (<70). Steroid duration, preoperative MHHS, and AVN severity were treated as explanatory variables.

Data were collected by a pre-tested, pre-formed semi-structured questionnaire by the corresponding author. All authors were in charge of cross-checking each and every questionnaire. Data were meticulously entered into statistical software and checked for inconsistencies, missing data, or wrong entries. Data were analyzed using SPSS Statistics version 25.0 (IBM Corp., Armonk, NY, USA). Descriptive statistics were presented as mean ± standard deviation (SD) for continuous variables and frequency (percentage) for categorical variables. The Mann-Whitney U test was applied to compare non-normally distributed continuous variables (e.g., steroid duration, time to first hip pain, postoperative MHHS) between moderate and severe COVID-19 groups. The Wilcoxon signed-rank test was used for within-group comparison of preoperative vs. postoperative MHHS scores. A p-value < 0.05 was considered statistically significant.

## Results

**Figure 1:**
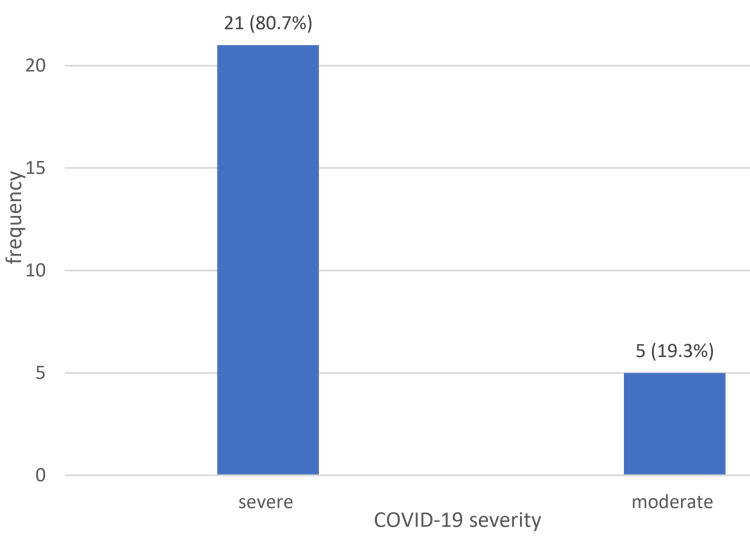
Distribution of the participants according to severity of COVID-19 infection (n= 26)

The study included 26 patients diagnosed with steroid-induced AVN of the femoral head. The mean age of the patients was 32.9 ± 6.3 years. Most patients were male (69.2%) (Table 1). Figure 1 illustrates that the majority of patients (80.8%) had a severe COVID-19 infection, while the remaining 19.2% had a moderate infection.

**Table 1:** Distribution of the participants according to sociodemographic characteristics and clinical data (n= 26) Data expressed as frequency, percentage, and mean±SD.

Variables		Frequency	Percentage
Age (years)	20-30	10	38.5
	31-40	11	42.3
	≥41	5	19.2
	Mean±SD	32.9 ± 6.3	
Sex	Male	18	69.2

In terms of steroid use, all moderate cases were subjected to oral steroids (72 to 80 mg for 14 to 21 days), and severe cases to oral (64 to 72 mg/day for 14 to 28 days) and injectable steroids (250 mg/day for five to seven days). Patients with severe COVID-19 received a significantly longer course of steroids (25.76 ± 3.52 days) compared to those with moderate infection (16.8 ± 3.83 days), with a statistically significant difference (p = 0.002). Additionally, the onset of first hip pain after cessation of steroid therapy occurred earlier in the severe group (54.1 ± 9.08 days) than in the moderate group (81.2 ± 3.83 days), which was also statistically significant (p < 0.001) (Table 2).

**Table 2:** Comparison of steroid duration and appearance of first hip pain between moderate and severe COVID-19 infection (n=26) Data presented as mean±SD, median (IQR); ^m^Mann-Whitney U test

Variables			COVID-19 severity		Mann-Whitney U test value	p-value
			Moderate (n = 5)	Severe (n = 21)		
Total steroid dose (mg)	Mean±SD		1248.8±296.05	2759.1±3	0.0	0.001^m^
	Median (IQR)		1120 (1008-1554)	2678 (2594-3094)		
Steroid duration (days)		Mean±SD	16.8±3.83	25.76±3.52	7.0	0.002^m^
		Median (IQR)	14 (14-21)	26 (26-28)		
First appearance of hip pain (days)		Mean±SD	81.2±3.83	54.1±9.08	0.0	<0.001^m^
		Median (IQR)	84 (77-84)	56 (45-63)		

Assessment of functional outcomes

By using the MHHS, the moderate COVID-19 group had a mean preoperative score of 39 ± 17.50, improving to 89.2 ± 3.35 postoperatively. In the severe COVID-19 group, the scores improved from 35.05 ± 12.73 preoperatively to 85.8 ± 6.47 postoperatively. Both improvements were statistically significant (p = 0.043 for moderate; p = 0.001 for severe) (Table 3).

**Table 3:** Comparison of mean MHHS between moderate and severe COVID-19 infection (n=26) Data presented as mean±SD and median (IQR); ^w^Wilcoxon Signed Rank test; ^m^Mann-Whitney U test MHHS: Modified Harris hip score

Variables		COVID-19 severity		p-value (U-value)
		Moderate (n = 5)	Severe (n = 21)	
Mean preoperative MHHS	mean±SD	39±17.50	35.05±12.73	0.648^m^ (45.5)
	Median (IQR)	41(55-22)	33 (45-23)	
Mean postoperative MHHS	mean±SD	89.2±3.35	85.8±6.47	0.469^m ^ (41.5)
	Median (IQR)	90 (92-82)	89 (91-82)	
p-value		0.043^w^	0.001^w^	

**Figure 2:**
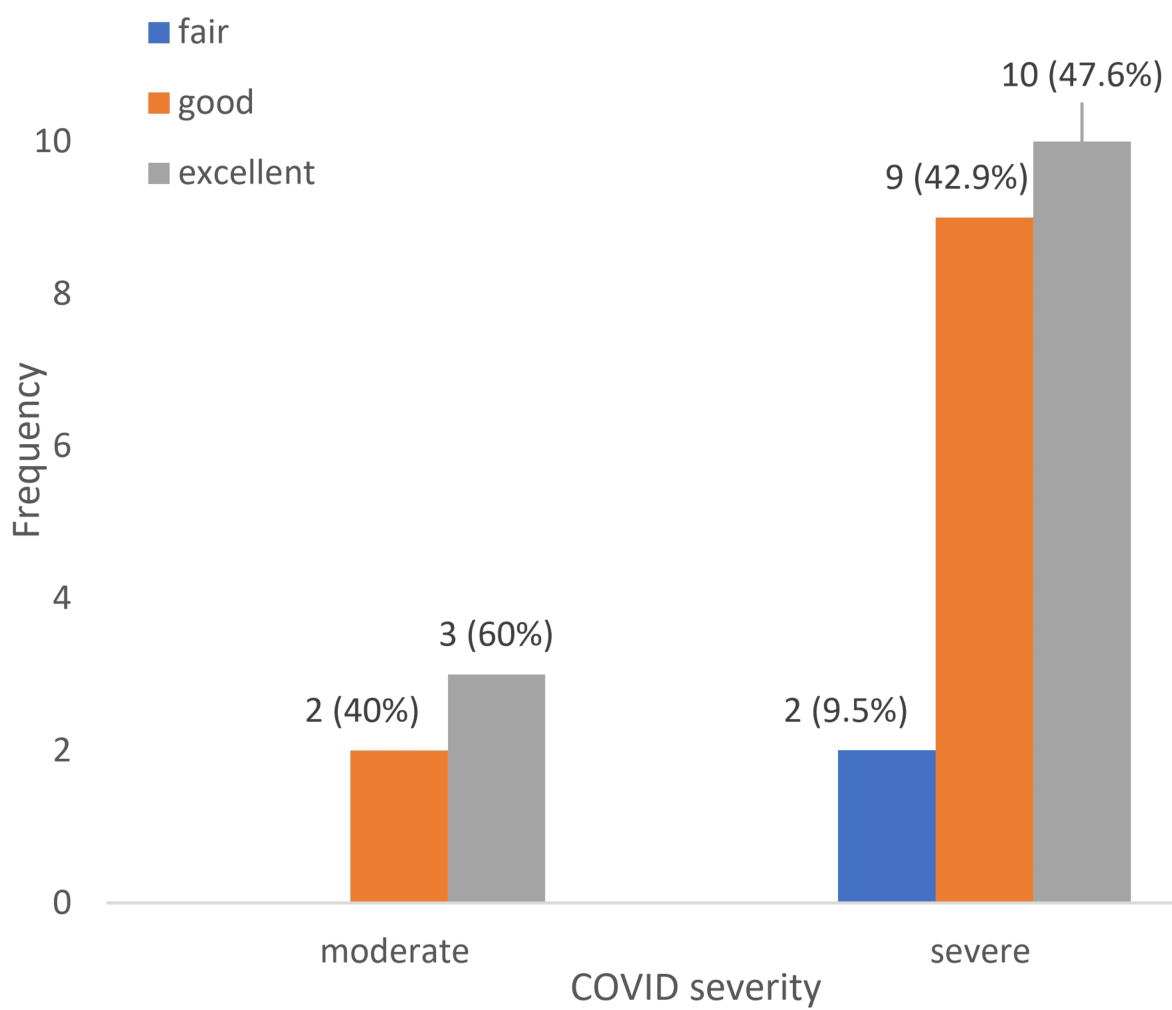
Comparison of postoperative functional outcome (n=26)

Figure 2 illustrates the comparison of postoperative functional outcomes between the moderate and severe COVID-19 groups. While both groups showed marked improvement, patients in the moderate group had a higher proportion of excellent outcomes postoperatively. No patient remained in the poor category (<70) after surgery. Steroid total dose, duration, and AVN severity showed no significant influence on functional outcome.

## Discussion

Corticosteroids are beneficial in treating moderate and severe COVID-19 cases, but long-term complications must be accounted for. Emerging evidence points toward a potential link between COVID-19 treatment with steroids and the development of hip AVN. The average age of steroid-induced AVN was about 33 years, and the male-to-female ratio was 2.33. Sakellariou et al. found the same age range of patients with male predominance [15]. This range is the active group of people who had to leave their households for work purposes.

In the present study, about 80% of patients with steroid-induced AVN had a history of severe COVID-19 infection, with an average duration of steroid intake of about 26 days, and it took an average of 55 days for hip pain to first appear after stoppage of steroid therapy. According to Velchov et al., 66.7% of the patients diagnosed with hip AVN had a history of severe COVID-19, and 33.3% had a moderate course of infection [16]. In the same study, it was revealed that the average duration of steroid administration was 30 days, and it took an average of 57.2 days for hip pain to appear. Banerjee et al. showed that patients presented with AVN features at a mean of 58 days after their initial COVID-19 diagnosis and treatment [17]. Although the literature is conflicting, both high-dose and cumulative steroid exposure, even over short periods, are recognized risk factors [18-20].

A significant improvement was seen in postoperative MHHS in both moderate and severe infections. However, the moderate group had significantly better functional recovery (p = 0.043) than the severe group (p = 0.001, both within-group comparisons). Per the MHHS, preoperatively, 26 patients (100%) were in the poor category. After surgical intervention, the moderate group reached a good to excellent functional outcome, and the severe infection group had 9.5% of patients with fairly good function.

Although patients in the moderate COVID-19 group appeared to achieve better postoperative hip function scores than those in the severe group, the difference was not statistically significant. This suggests that COVID-19 severity may influence recovery, possibly due to variations in steroid exposure, systemic inflammation, or physiological stress. Comparable findings have been reported in the literature: Kim et al. noted an improvement in mean HHS from 52.9 to 96 [21]; Celebi et al. reported a rise to 87.5; and Byun et al. documented a postoperative mean HHS of 98.2 [22,23].

Limitations 

This study has a small sample size, which may limit its generalizability. Despite our use of validated classification and scoring systems, reliance on convenience sampling and the absence of blinding during outcome assessment could nonetheless expose the study to both selection bias and observer bias. The 12-month follow-up, while adequate for short-term assessment, limits insight into long-term implant survival. We recommend conducting larger, multi-center studies for future research.

## Conclusions

While corticosteroids remain essential in managing moderate to severe COVID-19, their use must be approached with caution due to the risk of AVN. Despite significant functional improvement after THR in all cases, patients with moderate COVID-19 showed slightly better postoperative outcomes. The longer the duration of steroid use, the greater the dose, and the earlier the chance of development of hip necrosis. Patients should be counseled on this potential complication and provided with a detailed 'steroid card' for future reference. Early orthopaedic consultation and MRI screening are recommended for post-steroid joint symptoms to enable timely intervention. Total hip arthroplasty remains the preferred treatment in advanced AVN cases with joint destruction.
